# Diagnosis of latent tuberculosis infection among pediatric household contacts of Iranian tuberculosis cases using tuberculin skin test, IFN- γ release assay and IFN-γ-induced protein-10

**DOI:** 10.1186/s12887-021-02524-3

**Published:** 2021-02-11

**Authors:** Roxana Mansour Ghanaie, Abdollah Karimi, Leila Azimi, Seddon James, Mahshid Nasehi, Abolfazl Panahi Mishkar, Mahnaz Sheikhi, Fatemeh Fallah, Sedigheh Rafiei Tabatabaei, Seyedeh Mahsan Hoseini-Alfatemi

**Affiliations:** 1 Pediatric Infections Research Center (PIRC), Research Institute for Children’s Health (RICH), Shahid Beheshti University of Medical Sciences, Tehran, Iran; 2 Centre for International Child Health, Department of Paediatrics, Imperial College London, London, UK; 3 Center for Communicable Diseases Control, Ministry of Health and Medical Education, Tehran, Iran; 4 Department of Epidemiology and Biostatistics, School of Public Health, Iran University of Medical Sciences, Tehran, Iran; 5 Deputy of Health, Zabol University of Medical Science, Zabol, Iran; 6 TB Coordinator of Deputy Health, Golestan University of Medical Sciences, Gorgan, Golestan Iran

**Keywords:** Children, Interferon-γ release assays, Interferon-γ-induced protein-10, latent tuberculosis, Tuberculin skin test

## Abstract

**Background:**

Although the World Health Organization has recommended the diagnosis and prophylactic treatment of latent tuberculous infection (LTBI) in child household contacts of tuberculosis (TB) cases, the national programs in high-burden TB regions rarely implement adequate screening of this high-risk group, mainly because of resource limitations. We aimed to evaluate the prevalence of LTBI among pediatric household contacts of TB cases in two high-burden provinces in Iran.

**Methods:**

We conducted a cohort study in children who had been in household contact with a TB index. All subjects were assessed for active TB disease. For LTBI diagnosis, tuberculin skin test (TST) and QuantiFERON®-TB Gold Plus (QFT-Plus) were performed at the time of the index TB case diagnosis, as well as, 3, 12, and 18 months, if the first results were negative. In addition, interferon-γ-induced protein-10(IP-10) concentrations were measured for all participants.

**Results:**

A total of 230 children were enrolled, who had contact with an index TB case. Three contacts were diagnosed with active TB. According to the TST/QFT-Plus results, 104 (45.2%) children were identified with LTBI during our study. Significantly increased IP-10 levels were found in LTBI patients compared to healthy contacts. Accordingly, more than 50% of LTBI contacts and about 10% of healthy contacts were considered as IP-10-positive.

**Conclusion:**

This study alarmingly illustrates a high prevalence of LTBI among Iranian children exposed to TB cases. We, therefore, emphasize that the children living in close contact with an infectious TB case should be screened effectively and receive prophylactic therapy.

**Supplementary Information:**

The online version contains supplementary material available at 10.1186/s12887-021-02524-3.

## Background

Tuberculosis (TB), disease caused by *Mycobacterium tuberculosis* (Mtb), is one of the most important causes of childhood morbidity and mortality worldwide [1]. Globally, 1.1 million children under 15 years of age became ill with TB disease in 2018, and there were 205,000 deaths due to TB, according to the World Health Organization (WHO) reports [2]. Based on the global estimation, the incidence of TB is reducing by approximately 1.5% annually, however, this reduction will be insufficient for the elimination target set for 2050 proposed by the WHO [3]. This challenge could be due to the under-reporting and under-diagnosis of TB cases in many countries which has been estimated to be around one-third of the cases [4, 5]. Accordingly, to implement successful disease control strategies, the WHO has recommended the need for not only accurate diagnosis and treatment of active TB subjects but also the early diagnosis and treatment of latent tuberculous infection (LTBI) in the groups at high risk of developing a severe infection, particularly in the pediatric population [6, 7].

Children with LTBI do not show any symptoms of the active disease but could develop disease in the near or remote future, a process called TB reactivation [8]. Therefore, tracing and screening of pediatric household contacts of TB cases has a huge potential to detect and provide early treatment for children with TB and LTBI; however, although it is generally recommended by the WHO, it is infrequently practiced in resource-limited countries [9]. Young children with LTBI are at higher risk of developing active TB than adults, thus, it is of public health importance to identify LTBI in this population [10]. Moreover, in young children between 2 and 4 years of age, TB is often disseminated due to early, hematogenous spread of the mycobacteria after primary pulmonary infection and they have the highest risk of progression to central nervous system TB [11]. Most cases of progression to active TB disease among the pediatric population occur within 12 months of initial infection [12].

The tuberculin skin test (TST) and interferon-γ release assays (IGRA) are used for the screening of LTBI and are based on stimulating the cell-mediated immune system to detect whether it recognizes the antigens of Mtb [13, 14]. However, both TST and IGRA have reduced sensitivity in immunocompromised patients and cannot discriminate between active TB and LTBI [15]. Moreover, inadequate and insufficient investigations have been carried out in pediatric subjects, and these studies suggest that the performance of these assays varies from those obtained in adults, mainly in countries with a high incidence of TB [16]. Consequently, the diagnosis of LTBI should be qualified by the simultaneous use of various tools and measurement of other biomarkers to improve the sensitivity of the diagnosis. Based on these assumptions, the QuantiFERON®-TB Gold Plus (QFT-Plus), a new version of IGRAs, and the IFN-γ inducible protein 10 (IP-10) assay have been recently evaluated in the field of TB diagnosis [17, 18]. The QFT-Plus exploit both CD4+ and CD8+ T cells immune response to Mtb, having an important clinical value in conditions of immune depression due to CD4 T-cell impairments as in HIV-infection. In addition, a number of studies suggest that the CD8+ response is present at the onset of the infection and RD1-specific CD8 T-cells are more frequently detected during active TB compared to LTBI and, within LTBI, in recent infection compared with remote infection [19, 20]. IP-10 has been found to be increased in the plasma of TB patients and its advantage is that it is expressed in high levels following Mtb antigen-specific stimulation in both active TB and LTBI, suggesting its potential as a biomarker for TB in children [21, 22].

Although screening and management of pediatric household contacts of TB patients has great potential to reduce TB-related morbidity and mortality in children, the prevalence of LTBI cases among Iranian children has not been evaluated and reported so far. Therefore, the aim of the present study was to estimate the prevalence of LTBI among children exposed to TB cases and determine the accuracy of the IP-10assays for detection of children with LTBI.

## Methods

### Study design and population

This cohort study included a sample of children who had been in household contact with a TB index case within the previous 6 months. Case recruitment was performed in the regional reference centers for TB in Golestan and Sistan-Baluchestan provinces from July 2017 to August 2019.The list of adults with a history of cough > 2 weeks who had a diagnosis of sputum smear and/or culture-positive TB was provided. The adults with confirmed TB were questioned about any household contact with children under 18 years of age. Those responding positively were asked to bring the children to the selected health centers for evaluation and were invited to participate in our study. The study was undertaken at an out-patient level.

### Ethics statement

This study was approved by the Ethical Committee of Shahid Beheshti University of Medical Sciences, (approval number: IR.NIMAD.REC.1395.039). The patients or legal guardians of the participants provided signed informed consent forms for inclusion in the study.

### Radiological and microbiological evaluation

All child household contacts were clinically evaluated at the following times: at the time of the index TB case diagnosis, as well as, 3, 12, and 18 months, if the first results were negative. Chest X-ray was conducted in any contact with symptoms or signs of active TB and the clinical diagnosis of active pulmonary TB was made using a combination of clinical and chest X-ray features. All chest X-rays were examined by two pediatric pulmonologists, blinded to clinical details, and a scale of severity was determined as described by Petruccioli et al. They were classified as: 0: normal chest X-rays; 1: mild grade; 2: intermediate grade; 3: high grade [23]. Sputum/gastric lavage followed by smear examination and culture on Lowenstein Jensen media were performed for those with clinical/radiological features of pulmonary TB.

### TST and QFT-plus assays

QFT-Plus and TST were performed at the four times, as described above. The QFT-Plus assay was carried out according to the manufacturer’s instructions (QIAGEN, Germantown, MD, USA). Briefly, blood samples (4 ml) were collected and drawn directly into four QFT-Plus tubes (1 ml into each tube), TB1 tube containing the Mtblong peptide antigens derived from ESAT-6 and CFP-10 stimulating a cell-mediated immune response from CD4 T-cells; TB2tube with shorter peptides inducing CD8 T-cells in addition to the same antigens of TB1,Mitogen tube with non-specific mitogen phytohemagglutinin (PHA) to be used as the positive control and Nil (blank) tube to be used as the negative control. The three tubes were incubated at 37° for 16–24 h. Supernatant plasma was harvested from the tubes after centrifugation and was stored at − 80 °C. IFN-γ was measured using a QFT-Plus Analysis Software (www.quantiFERON.com) in a Bio-Rad (Hemel Hempstead, UK) plate reader (model 550) at 450 nm. The results were interpreted as negative, indeterminate, or positive using the manufacturer’s software. QFT tests were regarded as positive if the antigen-stimulated response of IFN- γ (TBAg-Nil) was 0.35 IU/ml or more, or negative if the mitogen stimulated response (Mitogen-Nil) was 0.5 IU/ml and the antigen-stimulated response was less than 0.35 IU/ml, or indeterminate if both mitogen-stimulated and antigen-stimulated responses were, 0.35 IU/ml and un-stimulated response (Nil) 0.8 IU/ml.

After QFT-Plus blood sampling, the two-step TST was performed by trained personnel following standard procedures. Accordingly, all participants were administered the TST via intradermal injection of 5 U of purified protein derivative (PPD) and indurations were measured using the palpation method 48–72 h later. TST results interpreted as positive (≥10 mm) and negative (< 10 mm).

LTBI was defined as the presence of a positive QFT-Plus result and/or positive TST result together with the absence of clinical signs/radiological evidence of active TB disease. In addition, in our study, the contacts without clinical and radiological evidence that were negative for both QFT-Plus and TST were considered as healthy contacts.

### IP-10 assay

IP-10 concentrations were measured in QFT-Plus supernatants using a Human IP10 ELISA Construction Kit (Antigenix America Inc., New York, NY) and classified as positive or negative according to a receiver operating curve as previously described by Yassin et al. [24].

### Statistical analysis

Data analysis was performed using SPSS v.22 (IBM Corp, New York, USA) and Prism 7 software (Graphpad Software 6.0, San Diego, USA). The ability of IP-10 to discriminate between children with and without TB infections was evaluated using Receiver Operating Characteristic (ROC) curves. ROC curves were constructed with LTBI contacts and healthy contacts. The significant area under curve (AUC) for LTBI and healthy contacts was compared to determine whether IP10 could distinguish between the two conditions. The Kruskall-Wallis and Mann-Whitney U tests were used for comparisons and a Bonferroni correction was applied. Statistical tests were considered significant if *P* values < 0.05.

## Results

### Study population

A total of 230 TB-exposed children were enrolled. Of these, 76 (33%) had been exposed in the last 6 months (e.g. retrospective cases) and 154 (67%) children were recruited prospectively as new index cases were diagnosed.

All the children enrolled had received the BCG vaccine in the first 2 months of life. Of the 230 children recruited, 52.1% were male and 47.9% were female. Moreover, the median age of the participants was 8.5 (range 1–17) years, and 4.8% were less than 24 months of age. Demographic characteristics and symptoms identified at the time of screening are shown in Table 1. Through clinical and microbiological evaluation, one household contact was diagnosed with active TB at baseline, one child 12 months after the diagnosis of the index case, and another 18 months after the diagnosis of the index case. Accordingly, 227 children (98.7%) remained asymptomatic.

**Table 1:** Demographic and clinical characteristics of the Iranian pediatric household contacts of TB cases

Demographic and clinical characteristics	N (%)
Adult tuberculosis source cases (*N* = 64)	
Sputum smear(+)/culture (+)	57 (89.1)
Sputum smear(−)/culture(+)	7 (10.9)
Household contacts < 18 years of age (*N* = 230)	
Male	120 (52.2)
Female	110 (47.8)
Age categories	
Age < 2 years	11 (4.8)
Age 2–5 years	38 (16.5)
Age 5–10 years	76 (33.1)
Age ≥ 10 years	105 (45.6)
Tuberculosis prophylaxis at the evaluation	11 (4.8)
Isoniazid preventive chemotherapy	7 (3)
Rifampin preventive chemotherapy	1 (0.4)
Ethambutol preventive chemotherapy	1 (0.4)
Pyrazinamide preventive chemotherapy	1 (0.4)
Other TB preventive chemotherapy	1 (0.4)
Symptoms at entry to the study	
Cough	5 (2.2)
Sputum	5 (2.2)
Weight loss	2 (0.9)
Fever	1 (0.4)
Chest pain	3 (1.3)

### TST and QFT-plus results

Our findings revealed that a total of 36% (80/230) of child TB contacts were TST positive at both baseline and follow-up. Exhaustively, 12.6% (29/230) of the evaluated contacts showed positive TST at baseline. Among contacts with negative baseline TST results,8% (16/201), 13.5% (29/185), and 8.3% (13/156) had become positive after 3, 12, and 18 months, respectively.

QFT-Plus results indicated that 20.9% (48/230) of child contacts showed positive IFN-γ responses at the baseline. Moreover, among the contacts with baseline QFT-Plus-negative results and valid follow-up QFT-Plus data, 13.7% (25/182), 6.4% (10/157), and 2.7 (4/147), showed an increase over the baseline IFN-γ value after 3, 12, and 18 months, respectively.

According to the TST/QFT-Plus results and the absence of clinical signs/radiological evidence of active TB, 45.2% (104/230) of children were identified as LTBI at the end of this study. Our findings revealed that 28.3% (65/230) of the household pediatric contacts were considered as LTBI at the baseline, while, the rate of LTBI conversion was 17% (39/230). Exhaustively, 2.3% (12/165), 14.4% (22/153), and 2.3% (5/131) of contacts were identified as LTBI after 3, 12, and 18 months, respectively. Table 2 shows the TST and QFT-Plus results among pediatric household contacts of TB cases.

**Table 2:** TST and QFT-Plus results in Iranian pediatric household contacts of TB cases

	TST+/QFT-	TST−/QFT+	TST+/QFT+	TST−/QFT-
At initiation of screening (*n* = 230)	18 (7.8%)	37 (16%)	11 (4.8%)	164 (71.3%)
3 months after the diagnosis of index (*n* = 164)	4 (2.4%)	14 (8.5%)	5 (3.1%)	141 (86%)
12 months after the diagnosis of index (*n* = 141)	16 (11.3%)	9 (6.4%)	1 (0.7%)	115 (81.5%)
18 months after the diagnosis of index (*n* = 115)	11 (9.6%)	3 (2.6)	1 (0.9%)	90 (85.8%)

Footnotes: *QFT-plus* QuantiFERON®-TB Gold Plus, *TB* tuberculosis, *TST* tuberculin skin test

### IP-10 results

**Fig. 1:**
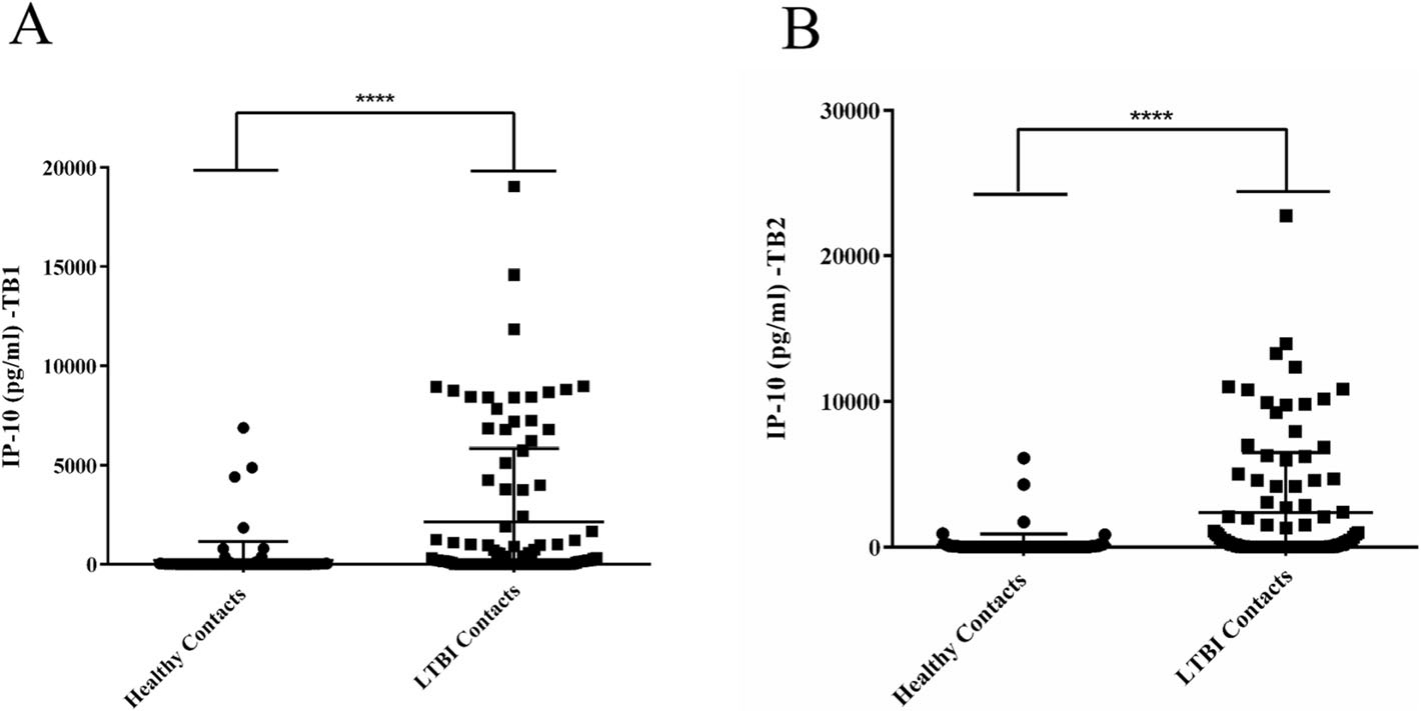
Significantly increased IP-10 levels in LTBI patients compared to healthy contacts. A) IP-10 levels in response toTB1 and B) TB2 stimulation. ELISA was carried out with QFT-Plus supernatants and IP-10 was expressed as pg/mL. The horizontal lines represent the median; statistical analysis was performed using the Mann-Whitney test with Bonferroni correction and **** represents *P*-value < 0.0001. Footnotes: IP-10: IFN-γ inducible protein10; LTBI: latent tuberculosis infection

Significantly increased IP-10 levels were found in LTBI patients (Mean ± standard error of the mean [SEM]: 2134 ± 362.5 for TB1 and 2379 ± 408.5 for TB2) compared to healthy contacts (TST−/QFT-) (Mean ± SEM: 208.6 ± 90.58 for TB1 and 158.4 ± 72.56 for TB2) in response to both TB1 and TB2 stimulation (Fig. 1). To define IP-10 results as positive or negative, a cut-off value for IP-10 was calculated using the ROC curve. For TB1 a cut-off of 67.2 pg/mL identifies LTBI with 60.2% sensitivity (50.5–69.1%) and 90% specificity (83.12–94.38%); similarly, for TB2, an IP-10 level > 142.3 pg/mL predicted LTBI with 53.4% sensitivity (43.8–62.7%) and 93.7% specificity (87.5–96.9%). Accordingly, 59.6% (62/104) of LTBI contacts and 11.3% (14/123) of healthy contacts were considered as IP-10-TB1-positive, respectively. In addition, the proportion of LTBI and healthy children with positive IP-10-TB2 was, respectively, 53.8% (56/104) and 8.9% (11/123).

## Discussion

Recognizing children with active TB or LTBI and either treating disease or targeting prophylaxis to high-risk groups are strategies recommended by the WHO in high TB burden regions. However, the identification of LTBI cases is more challenging in low–middle-income countries [25, 26].

To the best of our knowledge, this study is the most comprehensive investigation on the prevalence of LTBI among household child TB contacts in Iran. Our findings revealed that the prevalence and incidence of LTBI among Iranian children exposed to an index TB case with sputum smear and/or culture-positive adults were28.3 and 17%, respectively. These children are at high risk of developing active TB which is very worrying. Our findings are similar to other investigations in which only under half of child household contacts have evidence of LTBI [27, 28]. The high prevalence of LTBI found in our study supports that children are very susceptible to exposure to an active TB case and are at a high risk of LTBI [28]. Moreover, we detected three patients with active TB, in which two cases did not have clinical evidence of active disease at the beginning of the screening, thus were considered as incident cases. However, we cannot claim conclusively that the source of the infection in this population was the index case.

A meta-analysis including 95 studies from low- and middle-income settings estimated the prevalence of LTBI among household contacts to be 51.5% (95% CI 47.1–55.8%, I (2)=98.9%), with the highest incidence among children between 5 and 14 years of age [28]. In another study in Laos, Nguyen et al. reported that the prevalence of LTBI among child household contacts was 31.1% and increased with age from 26.0% in children below 5 years to 35.7% in children between 6 and 15 years [29]. So far, there is only limited data available on prevalence of LTBI among household contacts of TB cases in Iran. Previously, Shamaei et al. reported that 27.5% of close-family contacts of hospitalized TB patients in Iran were diagnosed with LTBI [30]. Therefore, our data reinforce the crucial importance of systematic screening and close follow-up of child household contacts, as well as a precise targeting of preventive treatment in this population.

TST is used as the only screening method to identify LTBI children in several high burden TB countries with limited resources, but the low sensitivity of TST, its poor specificity due to cross-reactions with environmental non-tuberculous mycobacteria and the BCG vaccine, as well as its unreliability in children with immunosuppression and malnutrition could limit the applicability of this approach [31, 32]. On the other hand, although IGRAs have emerged as promising alternatives to the TST, the limitations of these tests are gradually becoming apparent with their widespread use [33, 34]. For instance, previous surveys in both high and low TB burden regions confirmed that IGRA conversions and reversions could occur and there is some confusion about how to interpret such results [35–37]. Accordingly, several national guidelines (e.g. UK, Canada, Germany, the Netherlands, Italy, Switzerland, Norway, Spain, Ireland, and Korea) recommend a two-step approach of TST first, followed by an IGRA [33]. In the current study, we used both TST and IGRA assay to investigate LTBI cases among close contacts to an index TB case. In addition, we rechecked TST or IGRA in the subjects after 3, 12, and 18 months when the primary result was negative. Our findings indicated that there was adisagreement between TST and QFT-plus results. For contacts in whom TST and QFT-plus results were discordant, it is impossible to know which test was correct because there is no reference standard. In our study, BCG vaccination status might affect TST positivity in adjusted multivariable analysis. The quality of BCG vaccination among contacts and the percentage of subjects with booster vaccinations could contribute to the different degrees of concordance between TST and QFT-plus results. Moreover, although TST was performed by trained personnel following standard procedures, the disagreement between TST and QFT-plus results could be due to the variability in reading TST in the pediatric population. Some authors previously reported that the disagreement rate between TST and IGRA tests was about 10%, regardless of BCG vaccination [38, 39].

In the present study, we also evaluated the accuracy of IP-10 assay in the plasma of QFT-Plus for the diagnosis of LTBI among Iranian household pediatric contacts of TB cases. The results of some investigations suggest that IP-10 assay could be an alternative to IGRA, may be useful for diagnosing LTBI cases as it would be easier to use [18, 40]. Our findings indicated that IP-10 levels are significantly increased in contacts with LTBI compared to healthy contacts in response to both TB1 and TB2 antigens (*P value* < 0.0001). Considering the IP-10 results, no significant differences were observed between the TB1 and TB2. Therefore, the IP-10-based assay showed a high discriminating power and could help to distinguish between child contacts with LTBI from healthy contacts. In addition, a high concordance between the IP-10 assay and TST and QFT-plus tests was found.

IP-10 assay was previously thought to have significant advantages over IGRAs, including the possibility to use smaller blood volumes and to be detected in urine samples. It is also possible to measure IP-10 in dried plasma spots in filter papers allowing cheap and simple mail transportation at room temperature [18, 21, 41, 42]. Therefore, IP-10 assay would be useful as an uncomplicated and inexpensive alternative test for LTBI diagnosis in child household contacts of TB cases, especially in low-resource settings. Although in our studyIP-10 assay showed an acceptable specificity (90% for TB1 and 93.7% for TB2), it had a low sensitivity (60.2% for TB1 and 53.4% for TB2) for LTBI diagnosis. Importantly, it should be noted that the combination of the IGRAs and IP-10 assay has been reported to increase sensitivity [18]. Moreover, early studies suggest that the IP-10-based assay could not help to discriminate between active TB and LTBI among children [43].

The advantage of this study is that we used various approach for identification of LTBI among child household contacts, including TST, QFT, and IP-10 assay. Hence, our methodology allowed calculation of the prevalence of LTBI among child household contacts which, so far, has not been performed since LTBI cannot easily be demonstrated using a single method. However, some limitations could be noted in our study. For instance, investigator bias was further limited by the fact that TST could be interpreted differently by independent experts. Moreover, in this survey, some participants enrolled into the study retrospectively and others prospectively, which decreases the power of the study. Finally, in this investigation TST was performed by more than one investigator. Therefore, it might be cause pre-observer bias, which is difficult to objectively evaluate TST results.

## Conclusion

The results of this study alarmingly illustrate a high prevalence of LTBI among Iranian children exposed to an index TB case. Our experience, therefore, emphasizes that young children living in close contact with a source of TB should receive TB preventive therapy. In addition, establishing a national screening programme and incorporating various methods for LTBI identification among child household contacts should be taken into account in the design of TB control strategies in Iran. Importantly, the identification and prophylactic treatment of LTBI cases is crucial to the control and prevention of pediatric TB and requires more and greater efforts to this end. Moreover, the results of our study, if validated in further large-scale cohort studies, suggested that IP-10 assay could be an alternative biomarker for detection of Mtb infection and might provide some advantages over QFT and TST as the point-of-care test implementation.

## Supplementary Information

**Additional file 1.**

## Data Availability

The datasets supporting the conclusions of this article are included within the article.

## Abbreviations

BCG: Calmette-Gue’rin bacillus vaccine
CI: Confidence interval
IGRAs: Interferon- γ release assays
IP-10: Interferon-γ-induced protein-10
IQR: Interquartile range
HIV: Human immunodeficiency virus
LTBI: Latent tuberculous infection
Mtb: *Mycobacterium tuberculosis*
PHA: Phytohemagglutinin
PPD: Purified protein derivatives
QFT: QFT-Plus, QuantiFERON®-
TB: Gold Plus
TB: Tuberculosis
TST: Tuberculin Skin Test
WHO: World Health Organization
